# Knowledge of marine fish trematodes of Atlantic and Eastern Pacific Oceans

**DOI:** 10.1007/s11230-016-9629-9

**Published:** 2016-02-22

**Authors:** Rodney A. Bray, Pablo E. Diaz, Thomas H. Cribb

**Affiliations:** Department of Life Sciences, Natural History Museum, Cromwell Road, London, SW7 5BD UK; School of Biological Sciences, The University of Queensland, St Lucia, QLD 4072 Australia

## Abstract

A brief summary of the early history of the study of Atlantic Ocean marine fish digeneans is followed by a discussion of the occurrence and distribution of these worms in the Atlantic Ocean and adjacent Eastern Pacific Ocean, using the Provinces of the ‘Marine Ecoregions’ delimited by Spalding et al. (Bioscience 57:573–583, 2007). The discussion is based on a database of 9,880 records of 1,274 species in 430 genera and 45 families. 8,633 of these records are from the Atlantic Ocean, including 1,125 species in 384 genera and 45 families. About 1,000 species are endemic to the Atlantic Ocean Basin. The most species-rich families in the Atlantic Ocean are the Opecoelidae Ozaki, 1925, Hemiuridae Looss, 1899 and Bucephalidae Poche, 1907, and the most wide-spread the Opecoelidae, Hemiuridae, Acanthocolpidae Lühe, 1906, Lepocreadiidae Odhner, 1905 and Lecithasteridae Odhner, 1905. A total of 109 species are shared by the Atlantic Ocean and the Eastern Pacific, made up of cosmopolitan, circum-boreal, trans-Panama Isthmus and Magellanic species. The lack of genetic evaluation of identifications is emphasised and the scope for much more work is stressed.

## Introduction

The study of the marine trematode fauna of the world can be said to have started in the Atlantic Ocean basin, although the earliest recognisable post-Linnaean name to be coined for a marine digenean is probably *Fasciola ventricosa* Pallas 1774, now recognised as *Hirudinella ventricosa* (Pallas, 1774) Baird, 1853, a large stomach parasite of large scombrid fishes originally reported from Ambon Island, Indonesia but now reported worldwide (Pallas, 1774; Gibson, 1976). Other early descriptions which are still recognised include *Fasciola varicus* Müller, 1784, now known as the widespread and common species *Derogenes varicus* (Müller, 1784) Looss, 1901, originally reported from Danish waters (Müller, 1784). Carl Rudolphi in the early 19th Century made important early contributions, describing many worms from the Mediterranean Sea which are still recognised. Pérez-del-Olmo et al. (2016) considers these and the contributions of other workers in the Mediterranean. In the open Atlantic Ocean, Félix Dujardin (1845) described several species, such as those now recognised as *Cainocreadium labracis* (Dujardin, 1845) Nicoll, 1909, *Macvicaria soleae* (Dujardin, 1845) Gibson & Bray, 1982 and *Podocotyle angulata* Dujardin, 1845 off the Brittany coast, France. Other early workers in the north-eastern Atlantic include Thomas Spencer Cobbold (1858) who described the worm now recognised as *Lepidapedon rachion* (Cobbold, 1858) Stafford, 1904, presumably from a haddock apparently examined at Edinburgh, Scotland. Peter Olsson described many worms from the Scandinavian coasts (e.g. Olsson 1868) including many still recognised including *Steringophorus furciger* (Olsson, 1868) Odhner, 1905, *Zoogonoides viviparus* (Olsson, 1868) Odhner, 1902, *Zoogonus rubellus* (Olsson, 1868) Odhner, 1902 and *Fellodistomum fellis* (Olsson, 1868) Nicoll, 1909. Édouard van Beneden illustrated some worms from the Belgian coast (e.g. van Beneden, 1871) including those now recognised as *Steringotrema pagelli* (van Beneden, 1871) Odhner, 1911 and *Otodistomum cestoides* (van Beneden, 1871) Stafford, 1904.

Little was done in the Western or Southern Atlantic during the 19th Century. Joseph Leidy (1891) described a cercaria, *Distomum lasium*, from off New Jersey, USA, which is now recognised as *Zoogonus lasius* (Leidy, 1891) Stunkard, 1940. Edwin Linton started describing digeneans from various localities off the North American coast in the late 19th Century (e.g. Linton, 1889) and continued into the 20th Century with his most important work on the Tortugas of Florida (Linton, 1910) and a (posthumous) publication in 1940. Many of his species are still recognised including *Macvicaria crassigula* (Linton, 1910) Bartoli, Bray & Gibson, 1989, *Opechona pyriforme* (Linton, 1900) Bray & Gibson, 1990, *Opecoeloides vitellosus* (Linton, 1899) von Wicklen, 1946, *Prosorhynchoides arcuatus* (Linton, 1900) Love & Moser, 1983 and *Lintonium vibex* (Linton, 1900) Stunkard & Nigrelli, 1930.

Modern generic concepts began to be developed in the early 20th Century by Max Lühe and Arthur Looss in the Mediterranean and Teodor Odhner in the Atlantic. An important early work by the latter on Arctic worms (Odhner, 1905), was one of many by this author, many of whose species are still recognised in the genera in which he placed them, such as *Lecithaster confusus* Odhner, 1905, *Hemiurus levinseni* Odhner, 1905, *Aporocotyle simplex* Odhner, 1900, *Prosorhynchus aculeatus* Odhner, 1905, *Hemiurus communis* Odhner, 1905, *Proctophantastes abyssorum* Odhner, 1911 and many more. Other north-eastern Atlantic workers of the early 20th Century are the British workers William Nicoll and Marie Lebour, whose contributions include the recognised species *Fellodistomum agnotum* Nicoll, 1909, *Peracreadium idoneum* (Nicoll, 1909) Gibson & Bray, 1982, *Diphterostomum vividum* (Nicoll, 1912) Bray & Gibson, 1986, *Lepidapedon elongatum* (Lebour, 1908) Nicoll, 1910 and *Steringotrema ovacutum* (Lebour, 1908) Yamaguti, 1953.

In the early years of the 20th Century the northwestern Atlantic digeneans were studied notably by Joseph Stafford and Edwin Linton. Stafford (1904) described, but did not illustrate, worms from off the eastern Canadian coast. The fact that several are still recognised, e.g. *Homalometron pallidum* Stafford, 1904, *Neophasis pusilla* Stafford, 1904, *Stenakron vetustum* Stafford, 1904 and *Steganoderma formosum* Stafford, 1904, is due to the restudy of Stafford’s material by Max Miller (Miller, 1941).

No summary of the early work in the Atlantic Ocean would be complete without a mention of the contribution of Harold Manter. Starting in 1925 (Manter, 1925), he described many of the worms now recognised. There is no room to list them all, but they include *Dermadena lactophrysi* Manter, 1945, *Genolinea laticauda* Manter, 1925, *Gonocerca phycidis* Manter, 1925, *Multitestis blennii* Manter, 1931, *Megasolena hysterospina* (Manter, 1931) Overstreet, 1969, *Genolopa elongata* Manter, 1931, *Genolopa minuta* Manter, 1931, *Proctotrema lintoni* Manter, 1931 and *Prodistomum menidiae* (Manter, 1947) Bray & Gibson, 1990.

The study of the marine digenean fauna in the South Atlantic developed later. The first report in our database from the southwestern Atlantic is that of *Monorcheidespopovicii* Szidat, 1950 from off Tierra del Fuego (Szidat, 1950) and those from the south-eastern Atlantic are six monorchiids from off the coast of Ghana (Thomas, 1959).

Two other workers should be mentioned, due to their major contribution to the knowledge of digenean life-cycles in the Atlantic fauna. The first is Horace Stunkard who elucidated the life-cycle of *Zoogonoides laevis* Linton, 1940, *Proctoeces maculatus* (Looss, 1901) Odhner, 1911, *Stephanostomum dentatum* (Linton, 1900) Manter, 1940, *Opechona pyriforme*, *Lintonium vibex*, *Lepocreadium areolatum* (Linton, 1900) Stunkard, 1969, *Tubulovesicula pinguis* (Linton, 1940) Manter, 1947, *Lasiotocus minutus* (Manter, 1931) Thomas, 1959, *Neopechona cablei* Stunkard, 1980 and others (e.g. Stunkard & Uzmann, 1959). The other is Marianne Køie whose astonishing ability to coax digeneans into revealing their secrets allowed her to elucidate many life-cycles including those of *Opechona bacillaris* (Molin, 1859) Dollfus, 1927, *Zoogonoides viviparus*, *Stephanostomum caducum* (Looss 1901) Manter, 1934, *Monascus filiformis* (Rudolphi, 1819) Looss, 1907, *Steringophorus furciger* (Olsson, 1868) Odhner, 1905, *Derogenes varicus*, *Steringotrema pagelli*, *Fellodistomum fellis*, *Podocotyle reflexa* (Creplin, 1825) Odhner, 1905, *Aporocotyle simplex*, *Lepidapedon elongatum*, *Lecithaster gibbosus* (Rudolphi, 1802) Lühe, 1901, *Lecithochirium rufoviride* (Rudolphi, 1819) Lühe, 1901, *Hemiurus luehei* Odhner, 1905, *Lecithocladium excisum* (Rudolphi, 1819) Lühe, 1901, *Magnibursatus caudofilamentosa* (Reimer, 1971) Gibson & Køie, 1991, *Brachyphallus crenatus* (Rudolphi, 1802) Odhner, 1905 and *Hemiurus communis* Odhner, 1905 (e.g. Køie 1975, 1995).

## Database and methods

**Fig. 1:**
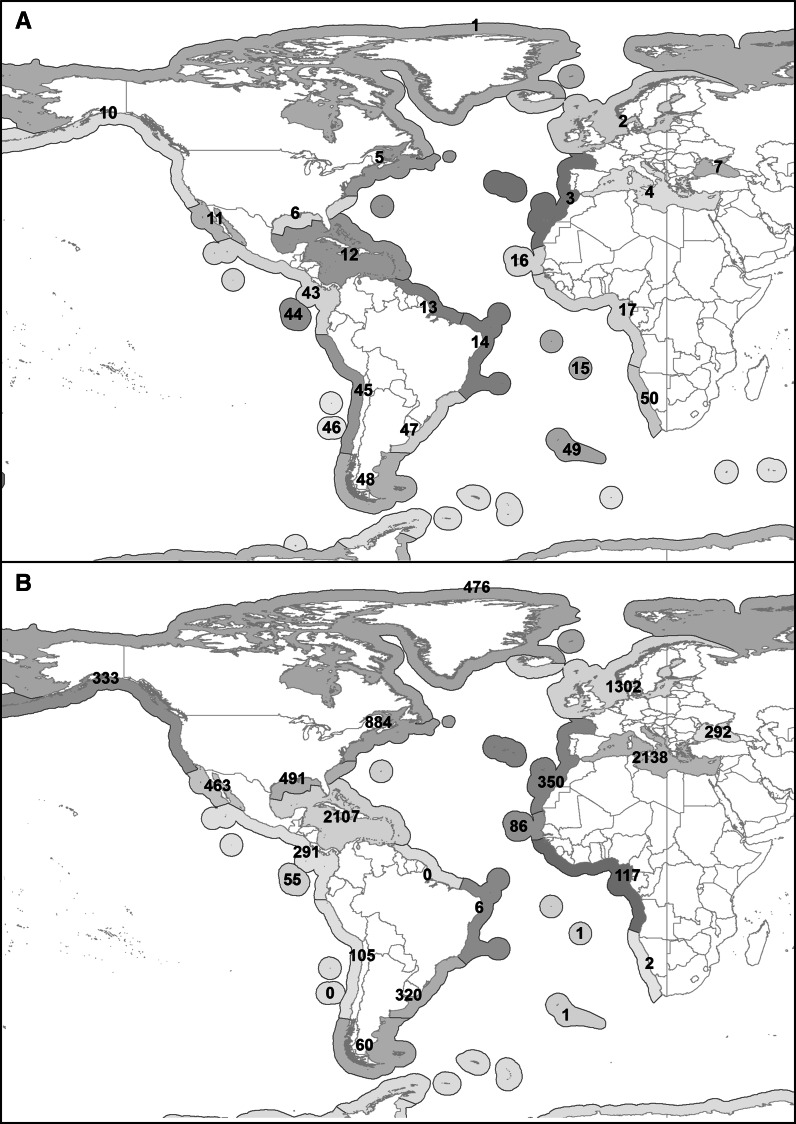
A, Map of the Atlantic and Eastern Pacific Oceans, showing ‘Spalding et al.’ Region Numbers; B, Map of the Atlantic and Eastern Pacific Oceans, showing the number of records (lines in the database) of digeneans in each Region

A database in the form of an Excel spreadsheet has been developed for the marine fish trematodes of the Atlantic Ocean. This is based predominantly on the work of one of us (THC) and his students, who have used the literature to write over 25,000 lines. The Atlantic and Eastern Pacific Ocean records from this database have been extracted and some further records have been added (by RAB and PED). The locality records have been coded according to Provinces of the ‘Marine Ecoregions’ delimited by Spalding et al. (2007) (see Table 1, Fig. 1A). These are ‘Large areas defined by the presence of distinct biotas that have at least some cohesion over evolutionary time frames’ (Spalding et al., 2007). Eastern Pacific Ocean Provinces are included for comparative purposes. The ‘Magellanic Province’ spans both Oceans as it includes the North Patagonian Gulfs, the Patagonian Shelf, the Falklands, the Channels and Fjords of Southern Chile and Chiloense. Overall data included in the survey include this Province in the discussion of the Atlantic fauna. The database suffers from the problems inherent in this type of enterprise, such as errors in entry, duplicates, omissions, typos (both in original reports and data entry), wrong attributions and unrecognised synonymies. Another problem is the lack of precision in the locality descriptions in many papers. These records have been omitted. Also omitted from the main analysis have been records of parasites and/or fishes which have not been identified to species. It has not been possible to consider the validity and synonymies of all the species, but in general the names follow that used in the World Register of Marine Species (WoRMS Editorial Board, 2015), compiled mainly by Dr David Gibson, and the series of ‘*Keys to the Trematoda*’ (Gibson et al., 2002; Jones et al., 2005; Bray et al., 2008).

**Table 1:** Atlantic and Eastern Pacific Provinces of the ‘Marine Ecoregions’ delimited by Spalding et al. (2007)

No.	Description
1	Arctic
2	Northern European Seas
3	Lusitanian
4	Mediterranean Sea
5	Cold Temperate Northwest Atlantic
6	Warm Temperate Northwest Atlantic
7	Black Sea
10	Cold Temperate Northeast Pacific
11	Warm Temperate Northeast Pacific
12	Tropical Northwest Atlantic
13	North Brazil Shelf
14	Tropical Southwestern Atlantic
15	St Helena and Ascension Islands
16	West African Transition
17	Gulf of Guinea
43	Tropical East Pacific
44	Galapagos
45	Warm Temperate Eastern Pacific
47	Warm Temperate Southwestern Atlantic
48	Magellanic
49	Tristan Gough
50	Benguela

## Results

Considering the number of records accumulated, it might be considered that we know a good proportion of the fauna, but the effort in different parts of the Ocean has certainly not been even.

The following data relating to the 17 regions of the Atlantic Ocean have been collated, along with the five Eastern Pacific regions: the number of species, genera and families in each region, the number of lines in the database per species, genus and family. These latter three parameters give an estimate of the effort in each region (Table 2). The number of lines in the database for each region is displayed on the Map (Fig. 1B).

**Table 2:** Number of lines in the database, species, genera, families and data on effort for the ‘Spalding et al.’ regions of the Atlantic and eastern Pacific Oceans

	Region	Lines	Species	Genera	Families	Lines per species	Lines per genus	Lines per family
Atlantic	1	476	66	46	16	7.2	10.3	29.8
	2	1,302	169	101	25	7.7	12.9	52.1
	3	350	126	86	26	2.8	4.1	13.5
	4	2,138	330	159	30	6.5	13.4	71.3
	5	884	160	93	26	5.5	9.5	34.0
	6	491	163	79	22	3.0	6.2	22.3
	7	292	85	58	17	3.4	5.0	17.2
	12	2,107	422	195	36	5.0	10.8	58.5
	13	0	0	0	0	0	0	0
	14	6	6	6	5	1.0	1.0	1.2
	15	1	1	1	1	1.0	1.0	1.0
	16	86	42	39	18	2.0	2.2	4.8
	17	117	56	45	17	2.1	2.6	6.9
	47	320	134	88	25	2.4	3.6	12.8
	48	60	22	18	10	2.7	3.3	6.0
	49	1	1	1	1	1.0	1.0	1.0
	50	2	2	1	1	1.0	2.0	2.0
Pacific	10	333	86	54	18	3.9	6.2	18.5
	11	463	139	98	26	3.3	4.7	17.8
	43	291	128	90	24	2.3	3.2	12.1
	44	55	33	24	11	1.7	2.3	5.0
	45	105	39	32	18	2.7	3.3	5.8

As can be seen, the Tropical Northwest Atlantic (12) has the most described species, followed by the Mediterranean Sea (4). In terms of lines per taxon, the Northern European Seas Province (2) is the most studied region, but the Arctic (1), Mediterranean Sea (4) and possibly the Cold Temperate Northwest Atlantic (5) and the Tropical Northwest Atlantic (12), have very similar levels of effort. The effort in the southwestern Atlantic (13, 14, 47, 48) is considerably less and no records were recovered from the North Brazilian Shelf Province (13). The best known fauna from this region is Warm Temperate Southwestern Atlantic (47). The south-eastern Atlantic (16, 17, 50) is the least studied continental coastal zone (with only two records recovered from the Benguela region −50). The two mid-Southern Atlantic Island provinces (15 and 49) have only one record each.

The database has in total 9,880 records of 1,274 species in 430 genera and 45 families, of which 8,633 are from the Atlantic Ocean, with 1,125 species in 384 genera and 45 families. About 1,000 species are endemic to the Atlantic Ocean basin, but Lessepsian migrants and other identifications of Indo-West Pacific species confuse the picture.

A major problem with interpretation of the database in this fashion is that it is reliant on accurate identifications. In addition, little effort has been made, hitherto, to explore the genetic validity of Atlantic species. Relatively recent works by, for example, Jousson (Jousson et al., 1999, 2000; Jousson, 2001), Blasco-Costa (Blasco-Costa et al., 2009a, b, 2010), Antar (Antar et al., 2015) in the Mediterranean and Curran, Pulis and Andres (Andres et al., 2014; Curran & Pulis, 2014; Pulis et al., 2014) in the Gulf of Mexico, are the exception. In the wider Atlantic the study of some deep-sea species of *Lepidapedon* Stafford, 1904 and other lepidapedids and fellodistomids (Lumb et al., 1993; Bray et al., 1994, 1999) has shown that cryptic species are common. New investigations should, wherever possible, include molecular evidence for the status of taxa.

Lack of precision in identification of parasites, i.e. those quote as ‘sp.’ or ‘spp.’, may be an indicator of the lack of taxonomic work in certain areas, where other specialists, e.g. ecologists, have been active. We have 66 such records from the Tropical Northwest Atlantic (Region 12) and 65 from the Mediterranean Sea (4) and the Warm Temperate Eastern Pacific (45). Considering the relatively few records of identified species in Region 45 (39% records are of unidentified worms), it seems clear that workers in this region, particularly in Chile and Peru, have suffered from a distinct taxonomic deficiency. On the other hand, Regions 12 and 4 are the most thoroughly studied regions and the number of records of unidentified parasites is relatively low (about 3% in both regions).

## The status of digenean families in the Atlantic Ocean

**Fig. 2:**
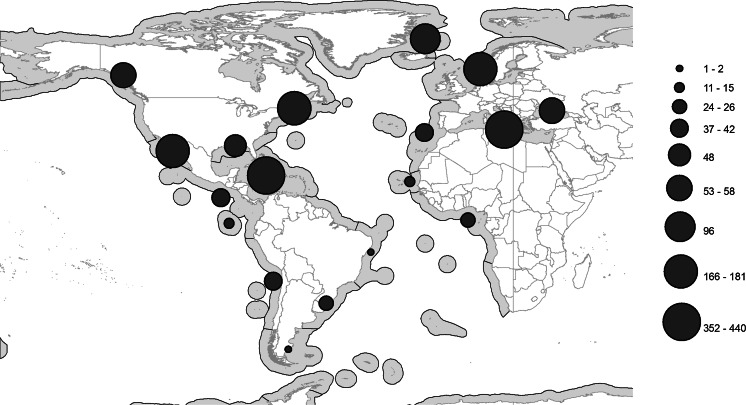
Map of the Atlantic and Eastern Pacific Oceans, showing the number of records (indicated by the size of filled circles) of Opecoelidae in each ‘Spalding et al.’ Region

**Fig. 3:**
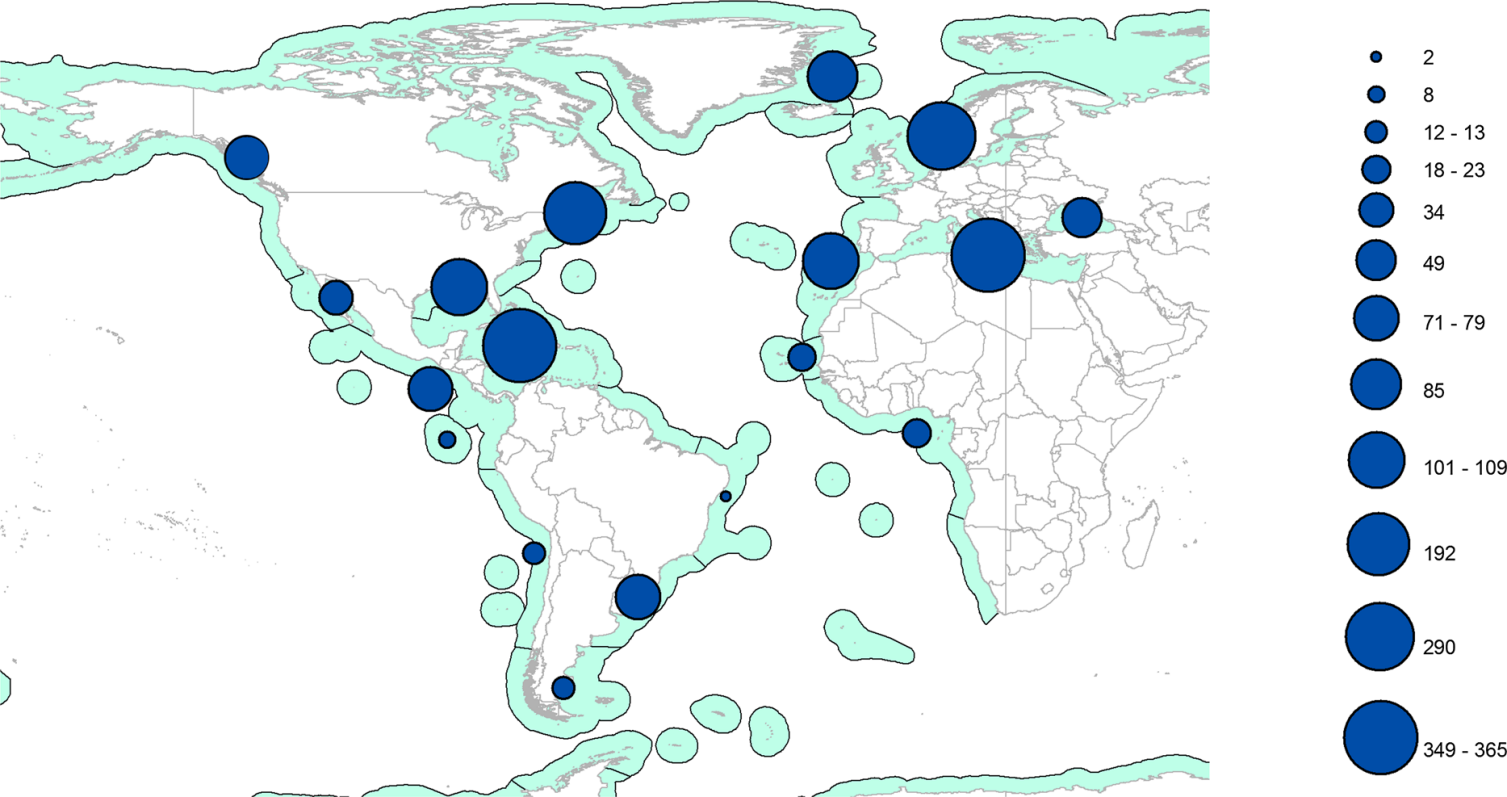
Map of the Atlantic and Eastern Pacific Oceans, showing the number of records (indicated by the size of filled circles) of Hemiuridae in each ‘Spalding et al.’ Region

**Fig. 4:**
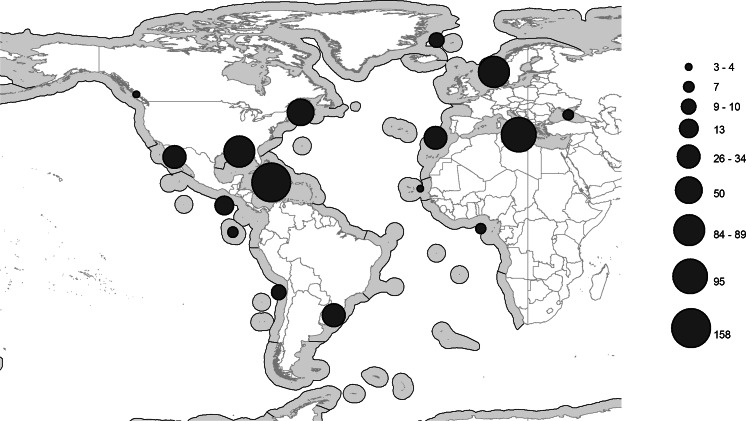
Map of the Atlantic and Eastern Pacific Oceans, showing the number of records (indicated by the size of filled circles) of Bucephalidae in each ‘Spalding et al.’ Region

The number of genera and species found in the families in the Atlantic Ocean are listed in Table 3, the distribution of records of the families in the Atlantic Ocean are listed in Table 4, and the distribution of reports of the three most species-rich families are illustrated in Figures 2–4.

**Table 3:** Numbers of genera and species in each family represented in the Atlantic Ocean

Family	No. of genera	No. of species	Family	No. of genera	No. of species
Opecoelidae Ozaki, 1925	50	160	Azygiidae Lühe, 1909	2	7
Hemiuridae Looss, 1899	32	101	Enenteridae Yamaguti, 1958	2	7
Bucephalidae Poche, 1907	11	92	Pronocephalidae Looss, 1899	5	6
Didymozoidae Monticelli, 1888	29	74	Syncoeliidae Looss, 1899	3	5
Acanthocolpidae Lühe, 1906	10	72	Hirudinellidae Dollfus, 1932	4	4
Lepocreadiidae Odhner, 1905	25	69	Gyliauchenidae Fukui, 1929	2	4
Monorchiidae Odhner, 1911	20	64	Lissorchiidae Magath, 1917	2	4
Fellodistomidae Nicoll, 1909	24	53	Diplangidae Yamaguti, 1971	1	4
Zoogonidae Odhner, 1902	19	40	Cladorchiidae Fischoeder, 1901	2	3
Lecithasteridae Odhner, 1905	13	40	Dictysarcidae Skrjabin & Guschanskaja, 1955	2	3
Cryptogonimidae Ward, 1917	18	38	Microphallidae Ward, 1901	2	3
Aporocotylidae Odhner, 1912	15	38	Bivesiculidae Yamaguti, 1934	1	3
Haploporidae Nicoll, 1914	13	35	Microscaphidiidae Looss, 1900	1	3
Derogenidae Nicoll, 1910	10	34	Opistholebetidae Fukui, 1929	1	2
Lepidapedidae Yamaguti, 1958	8	31	Bathycotylidae Dollfus, 1932	1	1
Apocreadiidae Skrjabin, 1942	10	29	Botulisaccidae Yamaguti, 1971	1	1
Haplosplanchnidae Poche, 1926	4	22	Brachycladiidae Odhner, 1905	1	1
Gorgoderidae Looss, 1899	6	19	Deropristidae Cable & Hunninen, 1942	1	1
Accacoeliidae Looss, 1899	8	15	Echinostomatidae Looss, 1899	1	1
Sclerodistomidae Odhner, 1927	6	10	Gorgocephalidae Manter, 1966	1	1
Faustulidae Poche, 1926	6	9	Opisthorchiidae Looss, 1899	1	1
Aephnidiogenidae Yamaguti, 1934	4	7	Ptychogonimidae Dollfus, 1937	1	1
Mesometridae Poche, 1926	4	7

**Table 4:** Number of regions in which families are represented

Families	No. of regions	Families	No. of regions
Hemiuridae Looss, 1899	13	Haplosplanchnidae Poche, 1926	5
Opecoelidae Ozaki, 1925	13	Ptychogonimidae Dollfus, 1937	5
Acanthocolpidae Lühe, 1906	12	Aephnidiogenidae Yamaguti, 1934	4
Lecithasteridae Odhner, 1905	12	Deropristidae Cable & Hunninen, 1942	4
Lepocreadiidae Odhner, 1905	12	Lissorchiidae Magath, 1917	4
Bucephalidae Poche, 1907	11	Bathycotylidae Dollfus, 1932	3
Derogenidae Nicoll, 1910	11	Gyliauchenidae Fukui, 1929	3
Fellodistomidae Nicoll, 1909	11	Mesometridae Poche, 1926	3
Monorchiidae Odhner, 1911	11	Dictysarcidae Skrjabin & Guschanskaja, 1955	2
Zoogonidae Odhner, 1902	11	Diplangidae Yamaguti, 1971	2
Cryptogonimidae Ward, 1917	10	Enenteridae Yamaguti, 1958	2
Apocreadiidae Skrjabin, 1942	9	Microscaphidiidae Looss, 1900	2
Didymozoidae Monticelli, 1888	9	Pronocephalidae Looss, 1899	2
Hirudinellidae Dollfus, 1932	9	Bivesiculidae Yamaguti, 1934	1
Sclerodistomidae Odhner, 1927	9	Botulisaccidae Yamaguti, 1971	1
Accacoeliidae Looss, 1899	8	Brachycladiidae Odhner, 1905	1
Aporocotylidae Odhner, 1912	8	Cladorchiidae Fischoeder, 1901	1
Gorgoderidae Looss, 1899	8	Echinostomatidae Looss, 1899	1
Haploporidae Nicoll, 1914	8	Gorgocephalidae Manter, 1966	1
Lepidapedidae Yamaguti, 1958	8	Microphallidae Ward, 1901	1
Syncoeliidae Looss, 1899	8	Opistholebetidae Fukui, 1929	1
Azygiidae Lühe, 1909	7	Opisthorchiidae Looss, 1899	1
Faustulidae Poche, 1926	7

The most species-rich, and jointly the most widespread, family is the Opecoelidae Ozaki, 1925 (Fig. 2), which is found in 13 of the 17 Atlantic regions. Considering that four of the zones are more or less completely unstudied, this presumably means that they are found in all zones. The second most species-rich family, the Hemiuridae Looss, 1899 has a very similar distribution pattern (Fig. 3). The third most species-rich family, the Bucephalidae, is found in fewer zones (11) than three less species-rich families (Fig. 4, Table 3). It is notable that the number of genera in the Bucephalidae Poche, 1907 is relatively few, but molecular results indicate that most of the currently recognised genera are polyphyletic (Nolan et al., 2015). Apart from the four more or less unstudied regions mentioned above (13, 15, 49 and 50), records of bucephalids are missing from the Magellanic (48) and the Tropical Southwestern Atlantic (14) regions. These are also poorly studied regions, and it is likely that bucephalids are, in fact, found in all regions.

The Atlantic is depauperate in some families that are relatively common in the Indo-West Pacific Region: the Gyliauchenidae Fukui, 1929, Enenteridae Yamaguti, 1958, Opistholebetidae Fukui, 1929 and Bivesiculidae Yamaguti, 1934 are notable examples. Other families are seemingly missing from the Atlantic entirely, including the Transversotrematidae Witenberg, 1944 and the Atractotrematidae Yamaguti, 1939.

These data emphasize again the paucity of work in some regions. The Lepidapedidae Yamaguti, 1958 is mainly a deep-sea family and the lack of deep-sea studies in the southern part of the Atlantic in particular is highlighted by its occurrence in only eight zones. On the other hand, the paucity of records of microphallids, echinostomatids and brachycladiids probably reflects the real situation. Microphallids are rarely found in fishes, but they do occur there occasionally (Siddiqi & Cable, 1960) and the occurrence of a brachycladiid in a shark is likely to be an accidental occurrence, although it was found in its usual site, the liver (Adams et al., 1998). The record of the ovigerous echinostomatid *Himasthla tensa* Linton, 1940 in the Atlantic cod *Gadus morhua* L. must also be considered an accidental infection (Linton, 1940).

## The Atlantic Ocean and the Eastern Pacific Ocean

According to our database 109 species are shared between the Atlantic Ocean and the Eastern Pacific region. These can be divided into four categories.

Fifty three of the species are cosmopolitan species. Some are parasites of widespread pelagic hosts, e.g. *Hirudinellaventricosa* and many didymozoids and others are frequently reported seemingly highly non-host-specific parasites, which may well represent cryptic complexes, e.g. *Helicometrina nimia* Linton, 1910, *Helicometra fasciata* (Rudolphi, 1819) Odhner, 1902 and *Derogenes varicus*. Recent studies (Calhoun et al., 2013) indicate that *H. ventricosa* is also a cryptic complex.

Thirteen species are circum-boreal, probably with a continuous population between the oceans *via* the Arctic Ocean (Table 5).

**Table 5:** Circum-boreal species, found in northern Atlantic and north-eastern Pacific regions

*Aporocotyle simplex* Odhner, 1900
*Brachyphallus crenatus* (Rudolphi, 1802)
*Genolinea laticauda* Manter, 1925
*Hemiurus appendiculatus* (Rudolphi, 1802)
*Hemiurus levinseni* Odhner, 1905
*Lecithaster gibbosus* (Rudolphi, 1802)
*Lecithophyllum botryophoron* (Olsson, 1868)
*Podocotyle atomon* (Rudolphi, 1802)
*Steganoderma formosum* Stafford, 1904
*Stenakron vitellosum* (Manter, 1934)
*Stephanostomum baccatum* (Nicoll, 1907)
*Steringophorus furciger* (Olsson, 1868)
*Zoogonoides viviparus* (Olsson, 1868)

Only four species appear to have been reported in the southern parts of both oceans, in the Magellanic region which is continuous around Cape Horn. These are *Aporocotyle ymakara* Villalba & Fernández, 1986, *Lecithochirium genypteri* Manter, 1954, *Neolebouria georgenascimentoi* Bray, 2002 and *Prosorhynchoides rioplatensis* (Szidat, 1970) Lunaschi, 2003. *Lecithochirium genypteri* is, however, circum-austral having been originally reported in New Zealand (Manter 1954), and later in Australian and South African waters (Korotaeva, 1975; Bray, 1991).

The most interesting group of shared worms is that found on either side of the Isthmus of Panama. Our database includes 38 such species (Table 6). Molecular exploration of the distinctions between apparent conspecifics on either side of the isthmus would allow calibration of evolutionary rates if (and when) consensus is reached on the time of the closing of the isthmus (Bacon et al., 2015). A case in point is the species listed as *Homalometron elongatum* Manter, 1947, which has been reported mainly in the Caribbean region (Manter, 1947; Sogandares-Bernal, 1959; Siddiqi & Cable, 1960; Fischthal, 1977; Bunkley-Williams et al., 1996; Parker et al., 2010), but also from two hosts and three localities in the Eastern Pacific (Sogandares-Bernal, 1959; Pérez-Ponce de León et al., 2007). Recent molecular work by Parker et al. (2010) has indicated that the Pacific form may well be a distinct, but genetically similar, species they named *H. lesliorum* Parker, Curran, Overstreet & Tkach, 2010. They considered ‘*H. elongatum* a widespread parasite of fishes throughout the Caribbean Sea, whereas *H. lesliorum* occurs in the Eastern Pacific Ocean off Central America’. There are some anomalies amongst the species in Table 6 in that as well as reports from the Eastern Pacific and western Atlantic, *Haplosplanchnus mugilis* Nahhas & Cable, 1964 (see Parukhin et al., 1971; Al-Bassel, 1997) and *Schikhobalotrema acuta* (Linton, 1910) (see Fayek et al., 1990) have both been reported from the Mediterranean Sea. It is likely that these are misidentifications.

**Table 6:** Species found on either side of the Isthmus of Panama

Trans-Panama Isthmus species	
*Aponurus pyriformis* (Linton, 1910)	*Lepidapedoides nicolli* (Manter, 1934)
*Cainocreadium oscitans* (Linton, 1910)	*Lepocreadium bimarinum* Manter, 1940
*Cardicola cardiocola* (Manter, 1947)	*Metadena globosa* (Linton, 1910)
*Cetiotrema carangis* (Manter, 1947)	*Myodera magna* Sogandares-Bernal, 1959
*Deontacylix ovalis* Linton, 1910	*Neolepidapedoides trachinoti* (Siddiqi & Cable, 1960)
*Dicrogaster fastigatus* Thatcher & Sparks, 1958	*Opecoeloides fimbriatus* (Linton, 1934)
*Haplosplanchnus mugilis* Nahhas & Cable, 1964	*Pachycreadium gastrocotylum* (Manter, 1940)
*Haplosplanchnus sparisomae* Manter, 1937	*Prosorhynchoides labiatus* (Manter & Van Cleave, 1951)
*Homalometron elongatum* Manter, 1947	*Prosorhynchus gonoderus* Manter, 1940
*Homalometron mexicanum* (Manter, 1937)	*Prosorhynchus ozakii* Manter, 1934
*Hymenocotta manteri* Overstreet, 1969	*Pseudoacanthostomum panamensis* Caballero, Bravo-Hollis & Grocott, 1953
*Hypocreadium biminensis* (Sogandares-Bernal, 1959)	*Pseudolepidapedon balistis* Manter, 1940
*Hypocreadium galapagoensis* (Manter, 1945)	*Pseudopecoelus priacanthi* (MacCallum, 1921)
*Hypocreadium scaphosomum* (Manter, 1940)	*Schikhobalotrema acuta* (Linton, 1910)
*Hysterolecitha brasiliensis* de Oliveira, Amato & Knoff, 1988	*Schikhobalotrema pomacentri* (Manter, 1937)
*Lasiotocus longicaecum* (Manter, 1940)	*Siphodera vinaledwardsii* (Linton, 1901)
*Lasiotocus truncatus* (Linton, 1910)	*Stephanostomum megacephalum* Manter, 1940
*Lecithochirium taboganus* (Sogandares-Bernal, 1959)	*Stephanostomum provitellosum* Sogandares-Bernal, 1959
*Lecithophyllum intermedium* (Manter, 1934)	*Stephanostomum tenue* (Linton, 1898)

The remaining species reported in both the Atlantic Ocean and the Eastern Pacific Ocean is *Tubulovesicula lindbergi* (Layman, 1930) Yamaguti, 1934, originally reported from Peter the Great Bay off North-eastern Russia (Layman, 1930), and well reported in both the western and the eastern North Pacific. The two reports from the Atlantic, from off Puerto Rico (Siddiqi & Cable, 1960) and Ghana (Fischthal & Thomas, 1972), are puzzling and probably represent misidentifications. This situation also illustrates the problems encountered in studies based on large databases, where it is not possible to verify every record.

## Lessepsian migration

The effect of man on the distribution of marine digeneans is exemplified by the effects of migration through the Suez Canal, so-called Lessepsian migration. We can be confident that this is changing the fauna, but to what extent it is not yet clear. For example, the herbivorous rabbit fish *Siganus rivulatus* Forsskål & Niebuhr has passed into the Mediterranean Sea, causing great damage to native algal assemblages (Sala et al., 2011), and bringing with it the wide-spread Indo-Pacific parasite *Thulinia microrchis* (Yamaguti, 1934) Bray, Cribb & Barker, 1993 (as *Hysterolecitha sigani* Manter, 1969) (see Fischthal, 1980; Bray et al., 1993). Also, recently, the cornetfish *Fistularia commersonii* Rüppell has spread right across the Mediterranean Sea along with its parasite *Allolepidapedon fistulariae* Yamaguti, 1940 (and other worms) (Pais et al., 2007). It is not yet known if these Lessepsian migrant parasites have spread into the open Atlantic Ocean. With the opening in August 2015 of a new channel parallel to the old one, the exchange of fauna between the Red and Mediterranean Seas is bound to increase (Galil et al., 2015).

## Concluding remark

Our data suggest 1,125 species in 384 genera and 45 families are reported in marine fishes of the Atlantic Ocean basin; almost 1,000 of these species are endemic to the Atlantic Ocean. The geographical region which is most poorly known is the South Eastern Atlantic Ocean, with few reports and, apparently, no current research. The deep-sea of the southern part of the Atlantic Ocean is also virtually un-studied.

The discovery of cryptic species in the Atlantic is in its early stages and will, without doubt, alter our understanding of the Atlantic digenean fauna as it has already in parts of the Indo-West Pacific (Miller et al., 2011). A molecular study of trans-Panama Isthmus species has the promise of estimation of speciation times and rates. There is much to be done, but modern techniques hold out much promise and could lead to an exciting future for workers in this Ocean.
